# Long-Term Outcomes of Active Surveillance and Immediate Surgery for Adult Patients with Low-Risk Papillary Thyroid Microcarcinoma: 30-Year Experience

**DOI:** 10.1089/thy.2023.0076

**Published:** 2023-07-13

**Authors:** Akira Miyauchi, Yasuhiro Ito, Makoto Fujishima, Akihiro Miya, Naoyoshi Onoda, Minoru Kihara, Takuya Higashiyama, Hiroo Masuoka, Shiori Kawano, Takahiro Sasaki, Mitsushige Nishikawa, Shuji Fukata, Takashi Akamizu, Mitsuru Ito, Eijun Nishihara, Mako Hisakado, Kazuyoshi Kosaka, Mitsuyoshi Hirokawa, Toshitetsu Hayashi

**Affiliations:** ^1^ Department of Surgery, Kuma Hospital, Kobe, Japan.; ^2^ Department of Head and Neck Surgery, Kuma Hospital, Kobe, Japan.; ^3^ Department of Internal Medicine, and Kuma Hospital, Kobe, Japan.; ^4^ Department of Diagnostic Pathology and Cytology, Kuma Hospital, Kobe, Japan.

**Keywords:** papillary thyroid microcarcinoma, active surveillance, immediate surgery, prognosis, disease progression

## Abstract

**Background:**

It has been 30 years since the initiation of active surveillance (AS) for adult patients with low-risk papillary thyroid microcarcinoma (PTMC). This study compared the long-term oncological outcomes of patients who underwent AS or immediate surgery (IS).

**Methods:**

This is a retrospective review of extended follow-up data from patients enrolled in a single-center, prospective observational study in Japan. In total, 5646 patients diagnosed with low-risk PTMC at Kuma Hospital between 1993 and 2019 were enrolled in this study. Of these, 3222 patients underwent AS (AS group), whereas 2424 underwent IS (IS group). The patients were followed up regularly, at least once per year. Descriptive outcome data were presented according to the treatment group.

**Results:**

In the AS group, 124 patients (3.8%) had tumor enlargement of ≥3 mm, and the 10- and 20-year enlargement rates were 4.7% and 6.6%, respectively. Novel lymph node metastases occurred in 27 patients (0.8%), and the 10- and 20-year nodal metastasis occurrence rates were 1.0% and 1.6%, respectively. In the IS group, 13 patients (0.5%) experienced lymph node recurrence postoperatively, and the 10- and 20-year nodal recurrence rates were 0.4% and 0.7%, respectively. Eighteen (1.4%) of the 1327 patients who underwent hemithyroidectomy experienced recurrence in the residual thyroid. The rate of lymph node metastasis was significantly higher in the AS group than in the IS group (1.1% vs. 0.4% and 1.7% vs. 0.7% at 10 and 20 years, respectively; *p* = 0.009), but the differences were small. However, the proportion of patients who underwent one or more and two or more surgeries was significantly higher in the IS group than in the AS group (100% vs. 12.3% and 1.07% vs. 0.09%, *p* < 0.01). Distant metastatic recurrence was observed in one patient after AS and conversion surgery and another after IS; however, they were alive (18.4 and 18.8 years after diagnosis, respectively). None of the patients in this study died of thyroid carcinoma.

**Conclusions:**

Long-term oncological outcomes of patients with PTMC generally did not differ clinically significantly between those undergoing AS and IS. AS is a viable initial management option for patients with low-risk PTMC.

## Introduction

The recent increase in the incidence of thyroid cancer in well-developed countries is mostly due to an increase in the detection of small papillary thyroid carcinoma (PTC).^1^ A PTC ≤10 mm without significant extrathyroidal extension, lymph node metastasis, or distant metastasis (T1aN0M0) is called a low-risk papillary thyroid microcarcinoma (PTMC). Active surveillance (AS) for such cancer was initiated at Kuma Hospital (Kobe, Japan) in 1993 after a proposal by Akira Miyauchi.^2,3^ In 1995, Sugitani et al. also initiated AS at the Cancer Institute Hospital (Tokyo, Japan).^4^ Favorable outcomes have been reported from these hospitals.^2–9^

With these promising outcomes, the Japan Association of Endocrine Surgery and the Japan Thyroid Association published consensus statements and a position paper, respectively, supporting its implementation.^10,11^ In 2016, the American Thyroid Association clinical practice guidelines for adult patients with thyroid nodules and differentiated thyroid cancer discussed consideration of AS as an alternative option to surgery for PTMC.^12^ Subsequently, many articles on AS were published from other countries.^13–18^ However, data on the long-term outcomes of PTMC managed with AS compared with surgery are needed. In this study, we enrolled 5646 PTMC patients who underwent AS or immediate surgery (IS) and compared their long-term outcomes.

## Materials and Methods

### Study design and patients

This is a retrospective review of data from a prospective study. In total, 5646 patients who were diagnosed with low-risk PTMC (T1aN0M0) using fine needle aspiration cytology (Bethesda V in 721 patients and Bethesda VI in 4925 patients) were enrolled in a prospective, observational cohort study at Kuma Hospital between October 1993 and December 2019. Patients with other thyroid malignancies and those <20 years of age were excluded from this study. Patients were offered two management options: IS and AS.

As previously reported in *Thyroid* in 2003, the study was approved by the hospital ethics committee and patients provided informed consent.^3^ The current retrospective extended follow-up study was approved by the ethics committee of Kuma Hospital (No. 20200709-1) and followed the revised guidelines of the Declaration of Helsinki (2013). Because of the retrospective nature of the present extended follow-up study, the requirement for additional informed consent was waived. The observational data in both groups were censored for statistical analyses in December 2022.

### Active surveillance

Patients who chose AS were asked to visit our clinic once or twice yearly for blood thyroid tests such as thyrotropin (TSH), free thyroxine (fT4) or free triiodothyronine (fT3), thyroglobulin and thyroglobulin antibody, neck ultrasound examinations, and levothyroxine prescription if patients reported hypothyroidism.^2,5,7–8^ Subsequently, a small number of physicians prescribed levothyroxine, with the patients' consent, to reduce serum TSH levels to low-normal or mild-sub-normal with the intention of preventing disease progression.^19^ Neck ultrasound was used to evaluate the tumor status and investigate the new appearance of suspicious lymph nodes. In the present study, the average maximum tumor size at the first and second examinations was set as the baseline tumor size to minimize observer variation as small fluctuations in tumor size were often observed during AS.^19^

If a tumor size increased ≥3 mm in any axis or in the maximum diameter compared with the baseline at two successive ultrasound examinations, the tumor was defined as enlarged at the point of the first ultrasound examination.^19^ Conversion surgery (CS) was recommended for patients exhibiting tumor enlargement. However, if the tumor location was not concerning, in the absence of other adverse features, and the patient wished to remain under AS, AS was permitted to be continued until the tumor reached 13 mm. When a suspicious lymph node was detected, a fine needle aspiration biopsy and cytological examination was performed, with measurement of thyroglobulin in the washout of the needle used for aspiration. If either of the results was positive for thyroid cancer metastasis, CS was recommended.

For patients who did not visit our clinic for a period of at least 18 months, we sent questionnaires asking about their current status and encouraged them to visit us if they were not followed up by other clinics.

### Postoperative follow-up

Patients who underwent IS or CS were asked to visit our outpatient clinic once or twice yearly for regular checkups, including tests similar to those of AS patients. On detection of a suspicious lymph node, evaluation was as described above. If a nodal metastasis was diagnosed, reoperation was recommended. For selected patients, chest computed tomography was performed at the attending physician's discretion. For patients referred to other hospitals or who did not visit our hospital, questionnaires were sent annually to evaluate their conditions. Their information regarding undergoing surgery, advanced disease status, and mortality was used in this study. Some included the data on tumor size. However, these values were not used in the current analysis as they may not be comparable with our data.

### Statistical analyses

StatFlex software (Artec, Osaka, Japan) was used to perform the statistical analyses. The comparison of clinicopathological features between groups was performed using chi-squared test or Fisher's exact test for categorical variables and Student's *t* test or the Mann–Whitney U test for continuous variables, as appropriate. The cumulative incidence of occurrence of lymph node metastasis was investigated using the Kaplan–Meier analyses, log-rank tests, and Cox proportional hazards regression analyses. Statistical significance was set at *p* < 0.05.

## Results

### Patients

**FIG. 1.**
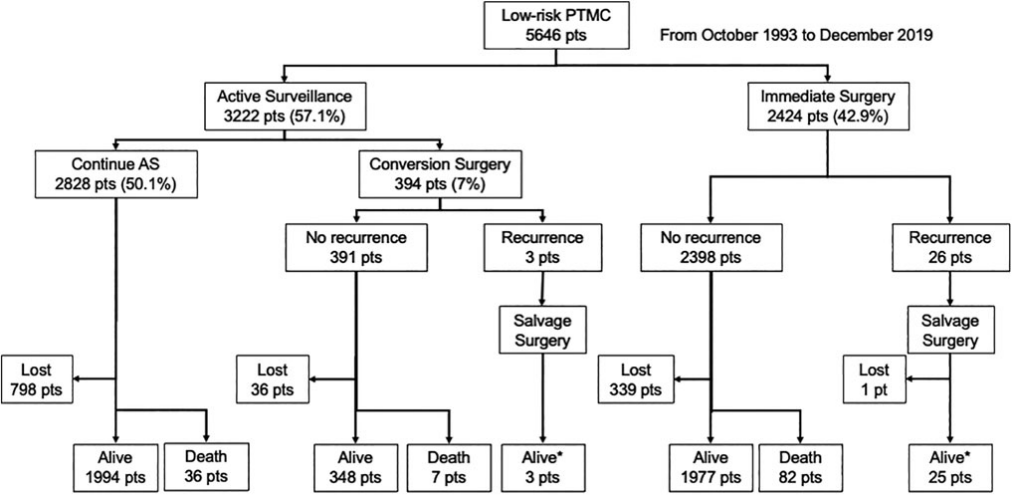
Patient flow diagram. Follow-up was censored in December 2022. Alive*, one patient each was alive with pulmonary metastasis; Death, all deaths were not due to thyroid cancer; Lost, lost to follow-up.

We included 5646 patients with PTMC, of which 3222 (57.1%) underwent AS for ≥1 year (AS group) and 2424 (42.9%) underwent IS within 1 year after diagnosis (IS group). A patient flow diagram is shown in Figure 1. Table 1 shows patients' characteristics in the AS and IS groups. The AS group was significantly older at diagnosis, had a smaller primary tumor, had multifocality less frequently, and was more often associated with Hashimoto's disease than the IS group (all *p* < 0.001). Of the included patients, only two (0.04%) had high-grade malignancy on cytology and underwent IS.

**Table 1.** Comparison of Patients' Backgrounds and Clinicopathological Features Between Active Surveillance and Immediate Surgery Groups

	AS group (%),* n* = 3222	IS group (%),* n* = 2424	*p*
Sex			
Male	398 (12.4)	240 (9.9)	0.004
Female	2824 (87.6)	2184 (90.1)	
Age at diagnosis (years)			
Median (range)	57.0 (20.0–92.0)	54.0 (20.0–92.0)	<0.001
Age <40 years			
Yes	408 (12.7)	370 (15.3)	0.005
No	2814 (87.3)	2054 (84.7)	
Age ≥60 years			
Yes	1406 (43.6)	858 (35.4)	<0.001
No	1816 (56.4)	1566 (64.6)	
Hashimoto disease^a^			
Present	1113 (34.5)	655 (27.0)	<0.001
Absent	2109 (65.5)	1769 (73.0)	
Graves' disease^b^			
Yes	216 (6.7)	189 (7.8)	0.115
No	3006 (93.3)	2235 (92.2)	
Family history of PTC^c^			
Yes	120 (3.7)	103 (4.2)	0.316
No	3102 (96.3)	2321 (95.8)	
Multiplicity^d^			
Yes	471 (14.6)	452 (18.6)	<0.001
No	2751 (85.4)	1972 (81.4)	
High-grade malignancy on cytology			
Yes	0 (0.0)	2 (0.04)	0.184
No	3222 (100.0)	2422 (99.9)	
Tumor size at diagnosis (mm)			
Median (range)	7.0 (2.0–10.0)	8.0 (2.5–11.5)	<0.001
Lost to follow-up before COVID-19			
Yes	572 (17.8)	506 (20.9)	0.003
Lost to follow-up in whole period			
Yes	834 (25.9)	340 (14.0)	<0.001
Period of follow-up (years)			
Median (range)	7.3 (1.0–29.3)	11.9 (1.1–29.3)	<0.001

Data are presented as numbers (percentages) or medians (ranges), as indicated.

^a^ Positive for antithyroglobulin and/or thyroid peroxidase antibodies.

^b^ Positive for anti-TSH receptor antibodies.

^c^ One or more first-degree relatives had PTC.

^d^ Evaluated using ultrasound examination.

AS, active surveillance; COVID-19, coronavirus disease; IS, immediate surgery; PTC, papillary thyroid carcinoma; TSH, thyrotropin.

However, the pathological diagnosis was conventional PTC without high-grade malignant features in both cases. The median duration of AS period in the AS group was shorter than that of postoperative follow-up in the IS group (7.3 years [1.0–29.3 years] vs. 11.9 years [1.0–29.3 years], *p* < 0.001). Before the coronavirus disease (COVID-19) pandemic, the proportion of patients lost during follow-up was significantly lower in the AS group (572/3222, 17.8%) than in the IS group (506/2424, 20.9%) (*p* = 0.003). However, as of December 2022, the percentage of patients lost to follow-up in the AS group was 25.9% (834/3222) and that in the IS group was 14.0% (340/2424).

### Clinicopathological features and outcomes of patients in the AS group who underwent CS and those in the IS group

In total, 394 patients (12.2%) in the AS group underwent CS for various reasons (Fig. 1). Table 2 shows the comparison of clinicopathological features and outcomes of patients in the CS and IS groups. There were no significant differences in sex and age, but the mean tumor size at surgery was 1 mm larger in the CS group than in the IS group (*p* < 0.001). The reasons for CS in the 394 patients were disease progression in 94 patients (23.9%), patients' preference in 58 (14.7%), others such as surgical indication for associated thyroid or parathyroid diseases in 79 (20.1%), and physicians' preference in 163 (41.4%). The extent of thyroidectomy and lymph node dissection was similar between both groups. However, an extrathyroidal extension was found significantly more often during surgery in the IS group than in the CS group (11.8% vs. 6.6%, *p* = 0.002).

**Table 2.** Comparison of the Backgrounds and Clinicopathological Features of Patients in the Active Surveillance Group Who Underwent Conversion Surgery with Those in the Immediate Surgery Group

	CS after AS (%),* n* = 394	IS group (%),* n* = 2424	*p*
Sex			
Male	38 (9.6)	240 (9.9)	0.928
Female	356 (90.4)	2184 (90.1)	
Age at surgery (years)			
Median (range)	55.0 (22.0–84.0)	54.0 (20.0–92.0)	0.193
Tumor size at surgery (mm)			
Median (range)	9.0 (3.0–19.0)	8.0 (3.0–18.0)	<0.001
Thyroidectomy			
Total thyroidectomy	188 (47.7)	1097 (45.3)	0.363
Hemithyroidectomy	206 (52.3)	1327 (54.7)	
Lymph node dissection			
Central+lateral	44 (11.2)	249 (10.3)	0.589
Central only	350 (88.8)	2175 (89.7)	
Extrathyroidal extension^a^			
Positive	26 (6.6)	286 (11.8)	0.002
Negative	368 (93.4)	2138 (88.2)	
pT status			
pT1a	336 (85.3)	2262 (93.3)	<0.001
pT1b	55 (14.0)	137 (5.7)	
pT3b	2 (0.5)	3 (0.1)	
pT4a	1 (0.3)	22 (0.9)	
pN status			
0	279 (70.8)	1666 (68.7)	0.436
1a	96 (24.4)	659 (27.2)	
1b	19 (4.8)	99 (4.1)	

Data are presented as numbers (percentages) or medians (ranges), as indicated.

^a^ Based on intraoperative findings.

CS, conversion surgery.

### Outcomes of PTMC in the AS group

**FIG. 2.**
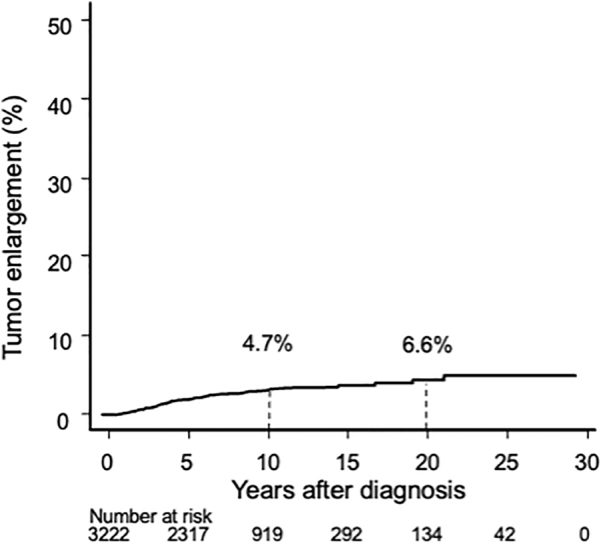
Kaplan–Meier curve of tumor enlargement rate in the AS group. AS, active surveillance.

**FIG. 3.**
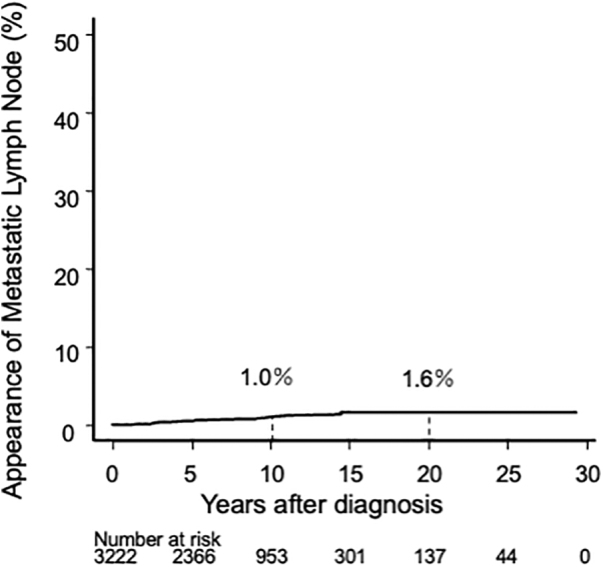
Kaplan–Meier curve of lymph node metastasis occurrence rate in the AS group.

In total, 124 patients (3.8%) in the AS group exhibited tumor enlargement, and the 10- and 20-year enlargement rates were 4.7% and 6.6%, respectively (Fig. 2). In the AS group, 27 patients (0.8%) showed a novel occurrence of lymph node metastasis during AS: 3 in the central compartment and 24 in the lateral compartment. The 10- and 20-year novel node metastasis occurrence rates were 1.0% and 1.6%, respectively (Fig. 3).

### Recurrence and occurrence of new PTMC lesions in the thyroid

**FIG. 4.**
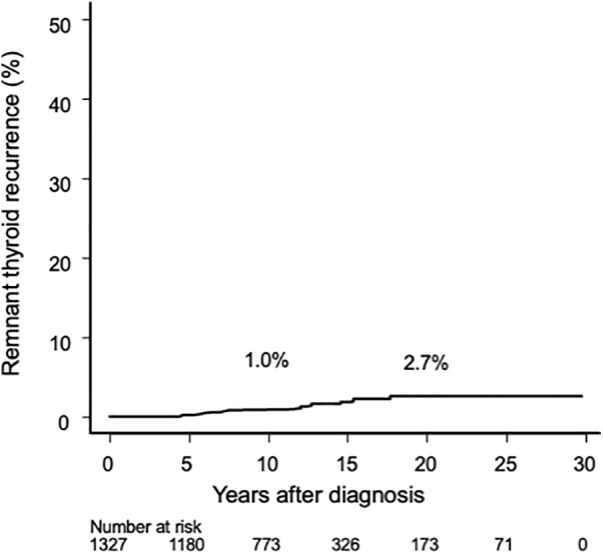
Kaplan–Meier curve of remnant thyroid recurrence rate in patients who underwent hemithyroidectomy in the IS group. IS, immediate surgery.

In the IS group, 1327 patients (54.7%) underwent hemithyroidectomy, and 18 (1.4%) showed recurrence or occurrence of new PTMC in the remnant thyroid, with 10- and 20-year rates of 1.0% and 2.7%, respectively (Fig. 4).

In the AS group, 13 patients (0.4%) were diagnosed with new additional PTMC lesions (8 in the contralateral lobe and 5 in the ipsilateral lobe or the isthmus). Eight patients underwent CS, whereas the remaining five remained under AS.

### Lymph node recurrence in the IS group

**FIG. 5.**
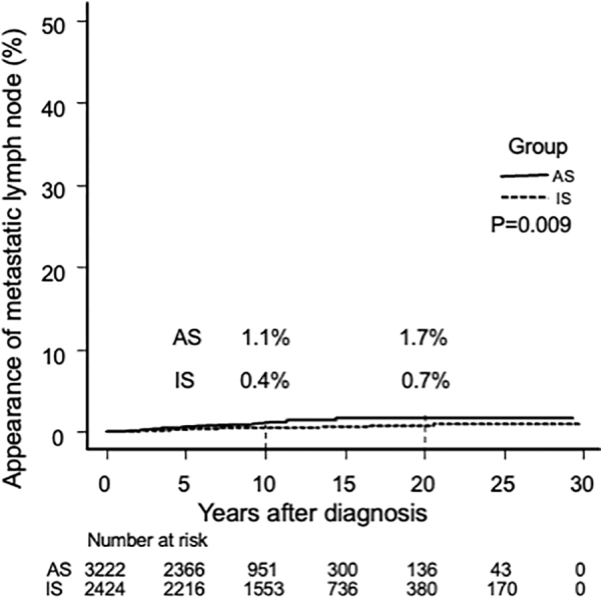
Kaplan–Meier curves of lymph node metastasis occurrence rate in the AS group and postoperative lymph node recurrence rate in the IS group.

In the IS group, 13 patients (0.5%) showed recurrence in the regional lymph nodes (1 in the central compartment and 12 in the lateral compartment), with 10- and 20-year node recurrence rates of 0.4% and 0.7%, respectively (Fig. 5).

We compared the cumulative incidence of nodal metastasis, including appearance during AS and recurrence after surgery in the AS group, with the postoperative node recurrence rate in the IS group. The cumulative incidence rate of nodal metastasis in the IS group was significantly lower than that observed in the AS group (*p* = 0.009: at 10 years, 0.4% vs. 1.1%; hazard ratio: 0.42 [confidence interval 0.20–0.87]) (Fig. 5). However, the risk difference was only 0.7% at 10 years and 1% at 20 years. Furthermore, 249 (10.3%) of the IS group patients underwent prophylactic lateral neck dissection at their initial surgery.

### Number of surgeries

All patients with metastatic lymph nodes required surgery. All the IS group had undergone surgical treatment after diagnosis, which included lateral neck dissection in 249 patients (10.3%). Of 3222 patients in the AS group, 394 had CS; in addition, 2 had surgery for lymph node recurrence, and 1 had resection of the remnant thyroid after hemithyroidectomy for a new PTC lesion (Fig. 1). Thus, this group had 397 surgical interventions in total (0.123 per patient) (Table 3). Of the 2424 patients in the IS group, additional surgical treatment was performed in 11 patients with lymph node recurrence, 11 patients with recurrence in the remnant thyroid after hemithyroidectomy, and 4 patients with local recurrence (Fig. 1). All patients in this group underwent surgery as the initial treatment. Thus, the total number of surgical interventions in the IS group was 2450 (1.012 per patient), and this was significantly larger than that of 397 (0.123 patient) in the AS group (*p* < 0.0001) (Table 3).

**Table 3.** Comparison of Numbers of Surgical Interventions: Active Surveillance Group vs. Immediate Surgery Group

No. of surgical interventions	AS group (3222 patients)	IS group (2424 patients)	*p*
0	2828 (87.8%)	0 (0%)	—
1	391 (12.1%)	2398 (98.9%)	<0.01
2	3 (0.1%)	24 (1.0%)	<0.01
3	0 (0%)	2 (0.1%)	—
Total number (/patient)	397 (0.123/patient)	2452 (1.012/patient)	

Proportion of patients who underwent one or more surgeries: 394 of 3222 (12.3%) in AS and 2424 of 2424 (100%) in IS (*p* < 0.01). Proportion of patients who underwent 2 or more surgeries: 3 of 3222 (0.09%) in AS and 26 of 2424 (1.07%) in IS (*p* < 0.01).

Of the AS group, 391 patients underwent 1 surgery and 3 patients underwent 2 surgeries (Table 3). Of the IS group, 2398 patients underwent 1 surgery, 24 underwent 2 surgeries, and 2 underwent 3 surgeries (Table 3). The proportion of the patients who underwent one or more surgeries was significantly higher in the IS group (2424/2424, 100%) than in the AS group (394/3222, 12.3%) (*p* < 0.01). The proportion of the patients who underwent two or more surgeries was also significantly higher in the IS group (26/2424, 1.07%) than in the AS group (3/3222, 0.09%) (*p* < 0.01).

### Occurrence of distant metastasis in the AS and IS groups

One patient in the AS group and another in the IS group showed developed metastasis. A 62-year-old female patient in the AS group underwent CS 2 years after AS by preference. Seven years later, the patient underwent a second surgery for lymph node recurrence. Lung metastasis occurred 12 years after the initial diagnosis. The metastatic lesions did not accumulate radioactive iodine; however, she was alive 18.4 years after the initial diagnosis. A 58-year-old female patient in the IS group underwent second and third surgeries because of lymph node recurrence 4 and 7 years after IS, respectively. Lung metastases occurred 12 years after IS; these did not accumulate radioactive iodine. She was alive with lung, bone, and brain metastases 18.8 years after IS.

### Death of patients in the AS and IS groups

None of the patients in the present study died of thyroid carcinoma, whereas 41 (1.5%) and 81 (3.9%) patients in the AS and IS groups, respectively, died of other causes.

## Discussion

In this study, we investigated the clinicopathological features and outcomes of low-risk PTMC patients who underwent AS or IS during the most recent 30-year period. This clinical trial of AS for low-risk PTMC was initiated at Kuma Hospital after the proposal by Akira Miyauchi in 1993, based on the following hypothesis: most PTMCs remain small, observation without IS will detect progressive tumors, performing surgery only for a minority showing modest disease progression will be sufficiently effective, and performing surgery for all PTMCs will result in more harm than good.^2^ We previously reported favorable oncological outcomes in our study participants who underwent AS.^5,8^ However, to the best of our knowledge, this is the first report directly comparing the long-term outcomes of AS and IS in PTMC patients during the same period at a single thyroid disease center hospital.

When the AS was initiated, paper medical charts were used. At Kuma Hospital, we adopted an electronic medical record system in February 2005, which has many advantages enabling detailed analyses such as tumor volume doubling rate and time-weighted detailed TSH score.^19,20^ Therefore, most recent reports on AS of PTMCs from Kuma Hospital reported only patients seen after 2005. In this study, we report fundamental long-term outcomes of AS and IS management in patients with PTMC who were seen since 1993.

Because this was not a randomized study, there were some biases between the AS and IS groups. The IS group had younger individuals, more men, those with larger tumors, and those who more often had multiple tumors than the AS group. The attending physician might have considered AS for patients with these features as more risky, especially for young patients with a long life expectancy.

In our recent studies including the present study, the average maximum tumor size at the first and second ultrasound examinations was set as the baseline tumor size. When a size increase of ≥3 mm compared with the baseline was detected in two consecutive ultrasounds, the tumor was regarded as enlarged at the first examination that showed enlargement. To minimize interobserver variations and consider fluctuations in tumor size, we used this definition from our recent study published in 2023.^19^ We believe that this definition allows for more reliable data analysis.

The median AS period in the AS group was shorter than that of postoperative follow-up in the IS group. This was because the proportion of patients who chose AS in the early study period was smaller than that in the later period. One might consider the failure to follow-up during AS. However, until the pandemic of COVID-19, the loss to follow-up rate in the AS group was lower than that in the IS group. We encouraged our patients to attend regular checkups, and we sent reminder mails to patients who did not show up for more than 18 months. During the pandemic, however, in the AS group, the number of patients lost to follow-up increased. About 60% of patients in the IS group were prescribed levothyroxine, whereas the vast majority of patients in the AS group were not. The AS group did not have much incentive to come to the hospital, especially in the difficult situation of the pandemic.

We have previously reported that PTMC in young patients (<40 years) was more likely to progress and that in older patients (≥60 years) was less likely to progress.^4,18^ A recent multi-institute study from Korea also reported that young age <30 years, male sex, and tumor size ≥6 mm were factors significantly associated with progression under AS.^18^ In our study, the incidence of recurrence or occurrence of new PTMCs in the remnant thyroid after hemithyroidectomy was low regardless of age, and the 10-year remnant thyroid recurrence rate was only 1.0%. In such cases, complete thyroidectomy can be easily performed; however, AS may be an alternative treatment strategy. Noda et al. reported that 84% of such lesions grew slowly, were stable, or even decreased in size.^21^ They also reported that no further recurrence was detected after thyroidectomy for the remaining 16% of tumors that grew moderately, and none of the patients died of thyroid carcinoma.^21^ We have also previously reported that tumor multiplicity was independently associated with novel nodal metastatic occurrence.^18^

We compared the nodal metastasis occurrence rate in the AS group with the nodal recurrence rate in the IS group. Patients with lymph node metastasis require surgical intervention, which is considered relatively easy from a surgical perspective if the lesion has not been dissected previously. The outcome of neck dissection for small metastatic lymph nodes is generally good. However, surgical intervention is a major unfavorable event for patients. The proportion of patients having one or more surgeries and the proportion of patients who had two or more surgeries were significantly higher in the IS group than those in the AS group (100% vs. 12.3% and 1.07% vs. 0.09%, respectively). Based on these data, it is expected that most patients would prefer AS over IS.

In the present study, one patient in the AS group who underwent CS showed distant metastasis 12 years later, and another in the IS group showed distant metastasis 15 years later. To the best of our knowledge, this is the first report of a patient who underwent AS and developed distant metastasis. However, both patients showed lymph node recurrence during the postoperative follow-up, indicating that their tumors were originally aggressive or had become aggressive. Tuttle et al. reported that of 483 patients who underwent AS, one developed a tumor that grew rapidly 4.7 years after AS.^22^ Although very rare, PTMCs acting aggressively or becoming aggressive may be found in large cohorts of low-risk PTMC patients. To date, no molecular markers that can discriminate aggressive PTMCs from others have been identified. *TERT* promoter mutations have been reported in aggressive PTC cases;^23,24^ however, they were reported to be useless in PTMC cases.^25–27^ These studies indicate that this event is extremely rare and cannot be completely avoided, regardless of management.

Surgery for PTMC is not difficult; however, Oda et al. reported that the incidences of temporary and permanent vocal cord paralysis, hypoparathyroidism, and the necessity for levothyroxine were significantly higher in the IS group than those in the AS group.^28^ One might consider the possible increase in surgical complications due to advanced disease in CS. Recently, Sasaki et al. reported similarly low rates of surgical complications in 242 patients with PTMC who underwent CS after AS and 1739 patients who underwent IS.^29^ They also reported that in the IS patients, permanent vocal cord paralysis occurred in 15 (0.9%), including accidental injury to the recurrent laryngeal nerve in 4 (0.2%).^29^ Kuma Hospital is a specialized hospital for thyroid diseases, and all surgeons are experts in thyroid surgery. If these surgeries had been performed by non-experts, the incidence would have likely been much higher. Furthermore, most of these unpleasant events could be mitigated through AS, supporting Miyauchi's hypothesis described above.

Under the Japanese medical insurance system, the 10-year total cost of IS was 4.1 times that of AS.^30^ Furthermore, several studies on patients' quality of life (QoL) have been published. These studies used questionnaire tools such as thyroid cancer-specific health-related QoL, the Hospital Anxiety and Depression Scale, and the State–Trait Anxiety Inventory to evaluate physical features, psychological status, and individual personality traits. Most studies reported better scores on physical features for AS patients than for IS patients.^31–34^ Regarding psychological issues such as cancer anxiety, the results have been controversial. Many studies, including those from our institution, showed that the rate of anxiety and depression was lower in patients who underwent AS than in those who underwent IS.^31–35^ Notably, 83% of the patients undergoing AS at Kuma Hospital replied that AS was the best choice for them.^36^

Our study has several limitations. First, this was a retrospective review of extended follow-up data from a prospective, nonrandomized study performed at a single thyroid disease center hospital. Although the fundamental inclusion and exclusion criteria and the indication for CS were decided from the beginning of the trial, the actual management modality depended on patients' preferences and attending physicians' discretion, resulting in some bias in the clinical and demographic features of both groups. Over the past 30-year period, physicians' confidence in AS and preference for management have gradually evolved with accumulating experience and evidence.^37^

This is a natural phenomenon in the implementation of novel management modalities. However, this is the largest cohort study of PTMC patients (*n* = 5646) with the longest term of AS (30 years). Variations in the quality of examinations, such as ultrasound, might have been small owing to this being a single-institution study. A patient's psychological state, such as cancer anxiety, can be greatly influenced by who sees the patient and how the disease and its management are explained. Unfortunately, none of the previous studies have investigated the relationship between physician characteristics and patients' psychological states. If a physician is not confident in the safety and superiority of the management, this will affect the patient.

## Conclusions

The oncological outcomes of patients who underwent AS and IS were generally not clinically meaningfully different. Considering factors such as unfavorable surgery events, medical costs, and patients' QoL, AS is a viable initial management option for low-risk PTMC.
